# Integrated analysis of zone-specific protein and metabolite profiles within nitrogen-fixing *Medicago truncatula-Sinorhizobium medicae* nodules

**DOI:** 10.1371/journal.pone.0180894

**Published:** 2017-07-10

**Authors:** Aaron J. Ogden, Mahmoud Gargouri, JeongJin Park, David R. Gang, Michael L. Kahn

**Affiliations:** 1 Molecular Plant Science Program, Washington State University, Pullman, Washington, United States of America; 2 Institute of Biological Chemistry, Washington State University, Pullman, Washington, United States of America; Centre National de la Recherche Scientifique, FRANCE

## Abstract

Symbiotic nitrogen fixation (SNF) between rhizobia and legumes requires metabolic coordination within specialized root organs called nodules. Nodules formed in the symbiosis between *S*. *medicae* and barrel medic (*M*. *truncatula*) are indeterminate, cylindrical, and contain spatially distinct developmental zones. Bacteria in the infection zone II (ZII), interzone II-III (IZ), and nitrogen fixation zone III (ZIII) represent different stages in the metabolic progression from free-living bacteria into nitrogen fixing bacteroids. To better understand the coordination of plant and bacterial metabolism within the nodule, we used liquid and gas chromatography coupled to tandem mass spectrometry (MS) to observe protein and metabolite profiles representative of ZII, IZ, ZIII, whole-nodule, and primary root. Our MS-based approach confidently identified 361 *S*. *medicae* proteins and 888 *M*. *truncatula* proteins, as well as 160 metabolites from each tissue. The data are consistent with several organ- and zone-specific protein and metabolite localization patterns characterized previously. We used our comprehensive dataset to demonstrate how multiple branches of primary metabolism are coordinated between symbionts and zones, including central carbon, fatty acid, and amino acid metabolism. For example, *M*. *truncatula* glycolysis enzymes accumulate from zone I to zone III within the nodule, while equivalent *S*. *medicae* enzymes decrease in abundance. We also show the localization of *S*. *medicae's* transition to dicarboxylic acid-dependent carbon metabolism within the IZ. The spatial abundance patterns of *S*. *medicae* fatty acid (FA) biosynthesis enzymes indicate an increased demand for FA production in the IZ and ZIII as compared to ZI. These observations provide a resource for those seeking to understand coordinated physiological changes during the development of SNF.

## Introduction

The largest contribution of nitrogen into the biosphere results from symbiotic interactions between legumes and soil bacteria of the family Rhizobiaceae [1–3]. This process, symbiotic nitrogen fixation (SNF), requires both plants and bacteria to drastically alter their metabolisms in order to establish the unusual conditions needed for SNF [4]. The developmental transition leading to SNF is initiated by nitrogen deficient legumes, which release flavonoids and other diffusible metabolites into the rhizosphere [5]. Rhizobia perceive these signals and respond by excreting Nod factors, lipochitooligosaccharides (LCOs) that interact with plant lysin-motif (LysM) receptors to stimulate the common symbiotic pathway (CSP) [6,7].

The CSP allows rhizobia to invade plant root hairs through tubular plant membrane channels called infection threads (ITs) [8]. Throughout symbiosis, ITs continue to traffic rhizobia into adjacent plant cells where they are released via endocytosis [9]. Endocytotic rhizobia are maintained within a plant-derived membrane, together referred to as the symbiosome, which prevents direct contact between symbionts. Within the infected plant cells, rhizobia begin a metabolic transition from bacteria to bacteroids, a differentiated state that is capable of fixing nitrogen [10].

As infection threads form, the CSP also initiates nodule organogenesis. For galegoid legumes, such as *Medicago truncatula*, nodule organogenesis involves the formation of a persistent plant meristem [11]. Persistence of a meristem has substantial consequences for both nodule structure and function. Most notably, the meristem produces a cylindrical mature nodule that contains five developmentally and physiologically distinguishable zones [12–15]. The first and smallest zone (ZI) is a thin layer of 2–5 cells at the tip of the meristem in which there are few rhizobia, typically contained in ITs [15]. Inward from ZI is the larger infection zone II (ZII), containing ITs and recently endocytized rhizobia. Immediately proximal to ZII is the interzone II-III (IZ), in which the bacteria have partially transitioned into terminally differentiated bacteroids, and the plant cells prepare to support nitrogen fixation by inducing proteins like leghemoglobin and glutamine synthetase, and establishing the needed microaerobic conditions in which O_2_ levels are of the order of 5–10 nM [10,16,17]. SNF is considered mature and most active in fixation zone III (ZIII). ZIII has a rhizobial population composed primarily of bacteroids and is the largest zone of the nodules studied in this work. ZIII is characterized by proteins involved in fixating and assimilating nitrogen, as well as oxygen binding and amino acid metabolism [18–21]. After five or more weeks of growth, a senescent zone, ZIV, appears in the region of the nodule proximal to the root [15,16].

Despite being considered metabolically distinct, the extent to which these zones differ metabolically is undefined. Previous attempts to understand how metabolism is coordinated as SNF develops have probed the physiology of whole-nodules, nodules differing in age, and nodules arrested at particular growth stages and have typically followed the presence of one or a few mRNAs or proteins [22]. These studies generally do not profile the many physiological changes that occur as the nodule tissue differentiates. Recently, Roux et al. (2014) generated transcriptomes of each zone by combining laser capture microdissection (LCM) and Illumina platform-based RNA-seq [23]. While Roux et al. (2014) provided a comprehensive resource of zone-specific transcriptional changes, our understanding of SNFs metabolic development requires a complementary analysis of proteins and metabolites during development.

We report here the use of nano-ultra performance liquid chromatography (UPLC) coupled to label-free mass spectrometry (MS) to catalogue the protein profiles of whole nodules, roots, and three developmental fractions of nodules formed in the *M*. *truncatula-S*. *medicae* plant-bacterial system. Furthermore, we use gas chromatography (GC) in line with MS to create primary metabolite profiles for the three sub-nodule fractions. Unlike RNA, metabolites and proteins cannot be amplified during analysis and thus more tissue is needed than can be easily obtained using LCM. This limitation was resolved by pooling the sections collected from multiple nodules prior to metabolite or protein extraction. Accurate and reproducible capture of the target zones during sectioning was made possible by using the drastic accumulation of pigmented leghemoglobin as a marker. Leghemoglobin is characteristically induced in the IZ [10,16,17] and the transition zone separates ZI and ZII from the nitrogen fixation ZIII. Our methodology was validated by comparing our data with previously described zone-specific biomarkers in closely related indeterminate nodule systems. Furthermore, the high reproducibility and clear statistical distinction between each fraction was shown using multivariate statistical analyses. After validation, we aim to elaborate the coordinated changes in central carbon, fatty acid, and amino acid metabolism between symbionts.

## Materials & methods

### Plant and bacterial growth conditions

*M*. *truncatula* A17 Jemalong seeds were scarified and sterilized prior to germination using a protocol adapted from [24]. Seeds were first soaked in concentrated H_2_SO_4_ for five min followed by five washes with sterile distilled H_2_O (sdH_2_O). Seeds were then incubated overnight at 4°C in a solution of 100 μg/ml ampicillin, 100 μg/ml kanamycin, and 10 μg/ml rifampicin. Antibiotics were removed the following morning with five sdH20 washes, and the seeds were spread across agar filled petri plates containing yeast mannitol broth [25,26]. Allowing the seeds to vernalize and germinate on rich media validates the sterilization steps, and seeds showing contamination were discarded. To synchronize germination, seeds were vernalized in the dark at 4°C for 48 hr, after which they were relocated to a germination chamber for 24 hr at 20°C. Seedlings were then transferred to sterilized trays containing perlite at a density of 50 seedlings/tray. Perlite trays were then inoculated with either plant nutrient solution (PNS) +3mM urea, PNS, or PNS containing rhizobia for positive, negative, and treatment conditions, respectively [27]. *S*. *medicae* used for plant inoculation was cultured at 30°C overnight in minimal mannitol broth supplemented with 0.05% NH_4_Cl, and added to PNS to a final optical density of 0.1. After inoculation, trays were transferred into insect exclusion tents (Bugdorm 2120F) and grown in a greenhouse at 25°C under a light/dark cycle of 16/8. Plants were watered as needed and fertilized three times a week with PNS or PNS + 3 mM urea for negative and positive controls, respectively.

### Tissue harvesting

Whole nodules, primary root, and each sub-nodule fraction was collected 30 days post inoculation (dpi) of *M*. *truncatula* with *S*. *medicae*. To isolate the three zones, each nodule was hand sectioned under binocular magnification into three parts using the distinct red color caused by leghemoglobin accumulation as a marker of the metabolic shift from non-fixing zones ZI and ZII (Fraction I), the early fixing/ interzone (Fraction II), and the mature fixation zone ZIII (Fraction III). Fraction I was isolated from the distal end of the nodule, approximately one millimeter before the tissue became red. To collect Fraction II, a second excision was made approximately 0.5 millimeters proximal to the initial site of leghemoglobin accumulation. Finally, Fraction III was collected by excising approximately five millimeters from the previous cut. The three Fractions therefore consisted of a white zone Fraction I, a pink zone Fraction II, and a dark red zone Fraction III. Samples were sectioned in this manner, immediately flash frozen in liquid nitrogen, and stored at -80°C prior to protein or metabolite extraction. Approximately 50 plants were used for each replicate resulting in ~50 mg of tissue in all cases prior to homogenization and extraction.

### Metabolite extraction and derivatization

All samples were homogenized by mechanical disruption via bead beating (Tissuelyser II Retsch MM400, Qiagen). Samples were returned to liquid nitrogen every 60 seconds to prevent degradation. Following homogenization, samples were extracted with 500 μL of a mixture of methanol, isopropyl alcohol, and water (5:2:2). Samples were then vortexed briefly, incubated for five min in a sonicating water bath (5510 R-MT, Branson), and centrifuged at 16,000 x g for five min. The supernatant was then collected and dried under vacuum without heat. The resulting sample was extracted using 500 μL of a 1:1 acetonitrile and H_2_O, briefly vortexed, and sonicated for five min. As before, the extracts were centrifuged, and the supernatant dried under vacuum. Derivatization was carried out by first incubating the samples at 30°C for 1.5 hr in five μL of 20 mg methylhydroxylamine hydrochloride (Sigma-M50400) in 500 μL pyridine. Each sample was then derivatized by adding 45 μL of N-methyl-N-trimethylsilylfluoroacetamide with 1% trimethylchlorosilane (Sigma-69478) and incubating at 37°C for 30 min. Samples were then transferred to GC vials and analyzed the same day.

### Protein extraction and digestion

Protein extraction was adapted from protocols provided by [28]. First, tissue was homogenized via bead beating (Tissuelyser II Retsch MM400, Qiagen). Each sample was then precipitated with 1.0 ml 10% TCA/acetone and centrifuged at 16,000 x g for 5 min at 4°C. The resulting pellet was then washed with 80% methanol with 0.1 M ammonium acetate and centrifuged at 16,000 x g for 5 min at 4°C. Finally, the pellet was washed with 80% acetone and pelleted at 16,000 x g for 5 min at 4°C. After air-drying the samples to remove residual acetone, each pellet was mixed thoroughly with 0.5 ml/0.1 g starting material of 1:1 phenol (Sigma-P4557)/sdH20. After centrifugation at 16,000 x g for 5 min, the lower phenol phase was transferred to a new tube. Protein was precipitated overnight at 4°C by adding 0.1 M ammonium acetate in methanol at a ratio of 5:1 methanol/phenol. Protein was then pelleted at 16,000 x g for 10 min, air-dried, and resuspended in 50 mM ammonium bicarbonate pH 7.5. Protein was quantified with a Qubit Protein Assay Kit (Life Technologies, Carlsbad, CA) in compliance with the manufacturer’s protocol. Disulfide bonds were reduced using 100 mM dithiothreitol (DTT) at a ratio of 1:10 DTT/sample volume and incubated at 60°C for 30 min. Cysteine bonds were then alkylated with 300 mM iodoacetamide at the same volume ratio for 30 min at room temperature. Finally, protein was digested using trypsin (Promega V511A) at a 1:50 ratio of trypsin/protein, and incubated at 37°C for 8 hr.

### GC-MS parameters and processing

The relative abundance of primary metabolites was determined by gas chromatography-time of flight mass spectrometry (GC-TOFMS) using a Pegasus IV (LECO, St. Joseph, MI). Separation was performed using an RXI-5Sil MS with Integra-Guard column (30m x 0.25 mm ID x 0.25 μm film thickness (Restek, GmbH, Bad Homburg, Germany). Column conditions were held initially at 50°C for 1 min following a ramp to 330°C at 20°C/min. All injections were 1 μL in splitless mode with helium as a carrier gas flowing at 1 ml/min. Finally, ChromaTOF software version 4.41 with LECO/Fiehn Metabolomics database was used for processing. Peak intensity values were normalized by the starting fresh weight of each sample. For statistical analysis, features with >50% missing values were omitted and the remaining missing values were imputed using the K-nearest neighbor method [29]. Values were then gLOG transformed according to Metaboanalyst [30]. These normalized data were also used in two-tailed t-tests assuming equal variance and ANOVA.

### LC-MS/MS parameters and processing

Nano-ultra performance liquid chromatography-ion mobility time-of-flight tandem mass spectrometry (nano-UPLC-IMS-TOFMS/MS) was used to evaluate the relative abundance of protein within each sample. Chromatographic separation was achieved by injecting 100 ng of protein into a Nanoacquity (Waters, Milford MA) reverse phase C18 column at a flow rate of 0.45 μL/min with a gradient of 5% ACN to 95% ACN in H_2_O with 0.1% formic acid over 85 min. The Nanoacquity was in line with a Synapt G2S (Waters, Milford MA) equipped with an electrospray ionization (ESI) source operating in positive ion mode. High energy MS/MS function included a transfer collision energy ramp from 15–50 eV. The lock mass using Leu-enkephaline was acquired every 30 seconds. Comprehensive databases for peptide identification were constructed using UniProtKB, Swissprot-Prot, and TrEMBL for both *M*. *truncatula* and *S*. *medicae* [31,32]. Data processing was performed using TransOmics V1.1 (Waters, Milford MA). Data filtering required that peaks appear in at least two of three biological replicates, i.e., 66.6% consistency and was subsequently normalized by total ion current (TIC). It is known that the population/proportion of rhizobia within the nodule increases from FI to FII and FIII. This became apparent as the contribution of bacterial peptide intensities to the TIC increased from FI to FII and FIII. We accounted for population differences by scaling the intensity of each feature such that their contribution to the TIC was equivalent across samples (S1 Table). For statistical analysis, features with >50% missing values were omitted from analysis, and the remaining missing values were imputed using K-nearest neighbor method [29]. Values were then gLOG transformed according to Metaboanalyst [30]. These normalized data were also used in two-tailed t-tests assuming equal variance and ANOVA.

## Results

### Highly distinct protein profiles of five plant and bacterial SNF tissues

**Fig 1:**
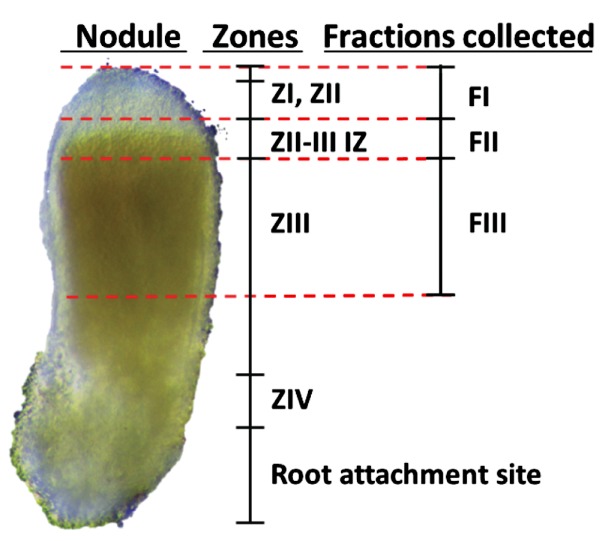
Zone capture method. A longitudinal section of a representative *M*. *truncatula-S*. *medicae* root nodule, with red lines indicate the location of incision when separating each zone. Also included in the analysis are whole nodules and primary root.

To evaluate the physiology of tissues associated with SNF, we developed a method for generating comprehensive protein profiles using label free nano-ultra performance liquid chromatography (nano-UPLC) coupled to mass spectrometry (MS). Tissues chosen for analysis included primary root, whole nodule, and three sub-nodule fractions of *M*. *truncatula* inoculated with *S*. *medicae* (Fig 1). Fraction one (FI) consisted of the colorless nodule tip, distal to the leghemoglobin accumulation indicative of IZ [10,16,17]. It is important to note that our FI contains both infection ZII and meristematic ZI. However, because ZII is much larger than ZI, our FI is likely representative of ZII, and we confirm this expectation later. The second fraction (FII) consists of an approximately 0.5 mm thick section taken at the site of leghemoglobin accumulation and represents the IZ. Fraction three (FIII) was taken from the center of the nodule proximal to the FII section, and represents the fixation ZIII. Finally, we also analyzed *S*. *medicae* inoculated primary roots collected 30 dpi.

Proteomic analysis of these five tissues identified 1,249 proteins with ≥2 unique peptides, consisting of 361 and 888 proteins mapping to *S*. *medicae* and *M*. *truncatula*, respectively (S2 Table). Table 1 displays examples of correlation between our fraction-specific MS data and previous localizations using mRNA hybridization, immunolocalization, proteomics, reporter gene fusion expression patterns, or transcriptomics [33–37]. For example, MtN1 (P93331_MEDTR) and ENOD 20 (MTR_4g130800) were significantly more abundant in FI compared to FII and FIII, a finding that is consistent with observations in the literature [38,39]. Furthermore, our FII protein levels agree with those previously described as IZ specific, including *M*. *truncatula's* nodule specific cysteine-rich peptide 333 (A7KHG3_MEDTR) and nodulin 22 (MTR_3g055450), as well as the nodule-specific glycine rich proteins 2A and 2B (MTR_2g042480 and MTR_2g042510, respectively). Finally, proteins known to be most abundant in nitrogen fixing zone III, including nitrogenase components (NifH), oxygen transporters (Lb1, MTR_5g066070), and calcium signaling machinery (MtCaML1, MTR_3g055570) are maximally accumulated in FIII. These results validate our FI, FII, and FIII as accurately representing ZII, IZ, and ZIII, respectively.

**Table 1:** Comparison of fraction-specific protein abundances with previously characterized zone-specific protein or transcript localizations.

Accession	Annotation	Published Pattern	Reference	%FI	%FII	%FIII
*M*. *truncatula*						
MTR_ 4g120950	MtN13	Nodule Apex	Gamas et al. 1998	48	27	26
P93331_MEDTR	MtN1	ZII	Gamas et al. 1998	80	13	7
MTR_4g130800	ENOD20	ZII	Vernoud et al. 1999	74	23	3
MTR_0093s0090	ENOD16	ZI & Symbiosome	Catalano et al. 2004	82	18	0
MTR_8g097320	SYMREM	ZII-III	Lefebvre et al. 2010	23	48	30
MTR_3g055440	MtN25	Symbiosome	Catalano et al. 2004	5	50	45
MTR_3g055570	MtCaML1	IZ-ZIII	Liu et al. 2006	0	21	79
MTR_7g078210	HSP70	Symbiosome	Catalano et al. 2004	4	52	45
MTR_5g066070	LB1	IZ-ZIII	de Billy et al. 1991	0	12	88
I3SJC8_MEDTR	AAT	ZII-ZIII	Yoshioka et al. 1999	1	14	85
MTR_3g055450	MtN22	IZ	Roux et al. 2014	15	56	28
MTR_2g042480	NGR 2A	IZ	Roux et al. 2014	15	67	19
MTR_2g042510	NGR 2B	IZ	Roux et al. 2014	7	89	4
MTR_4g033900	NCR	IZ	Roux et al. 2014	4	53	43
MTR_5g014710	MDH	IZ-ZIII	Roux et al. 2014	5	49	45
A7KHG3_MEDTR	NCR333	IZ	Roux et al. 2014	0	84	16
*S*. *medicae*						
Smed_0587	SOD	ZIII	Queiroux et al. 2012	6	32	62
Smed_6225	NifH	IZ-ZIII	Labes et al. 1993	20	24	56
Smed_1389	GS	ZI-ZIII	Roux et al. 2014	37	17	46
Smed_3389	DnaK	ZII-IZ	Roux et al. 2014	30	40	30
Smed_2941	AKGDH	ZII-IZ	Roux et al. 2014	56	28	16
Smed_1241	GyrA	ZI-IZ	Roux et al. 2014	77	20	3
Smed_1962	IlvC	ZI-ZII	Roux et al. 2014	70	18	12
Smed_0745	3OAR	ZI-IZ	Roux et al. 2014	74	19	8
Smed_3214	SecB	ZII-IZ	Roux et al. 2014	71	27	2

FI, Fraction I; FII, Fraction II: FIII, Fraction III; ZI, Zone I; ZII, Zone II; IZ, Interzone; ZIII, Zone III

**Fig 2:**
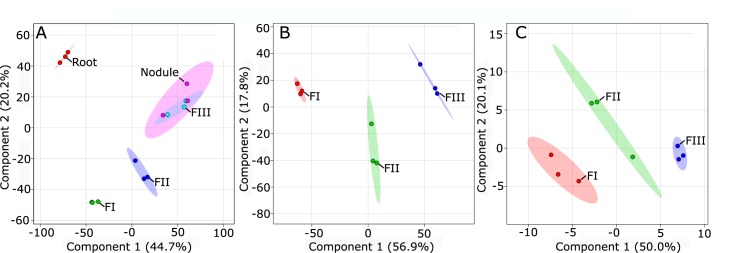
Multivariate analysis. (a) Scoreplot results from PCA using protein profiles of all tissues sampled. (b) PLS-DA scoreplot of fraction-specific protein profiles. (c) PLS-DA scoreplot of fraction-specific metabolite profiles. In all cases dashed circles indicate a 95% confidence region. FI, Fraction I; FII, Fraction II; FIII, Fraction III.

We hypothesize that previous experiments that collected protein profiles of whole-nodule lysates were in fact sampling the largest zone in the nodule, ZIII, which corresponds to our FIII. We also sought to evaluate whether the protein profiles of our FIII samples would be statistically distinguishable from the whole-nodule sample by principal component analysis (PCA). Fig 2A shows the PCA scoreplot generated from the protein profiles of each sample. Component 1 comprises 44.7% of data variation and is caused by differences between FI, FIII, and root samples, while component 2 shows 20.2% of data variation is predominately caused by differences between the root, FII, and FIII. The only tissues with protein profiles that did not differ substantially were FIII and the whole nodule, which is consistent with the fact that most of the nodule was composed of FIII. These observations also confirm our ability to reproducibly capture tissues with distinct protein profiles using our sectioning method. Analysis of each the three sub-nodule fractions alone demonstrates the majority of data variation was caused by differences between FI and FIII with intermediate values in FII at both metabolite and protein level (Fig 2B and 2C).

**Fig 3:**
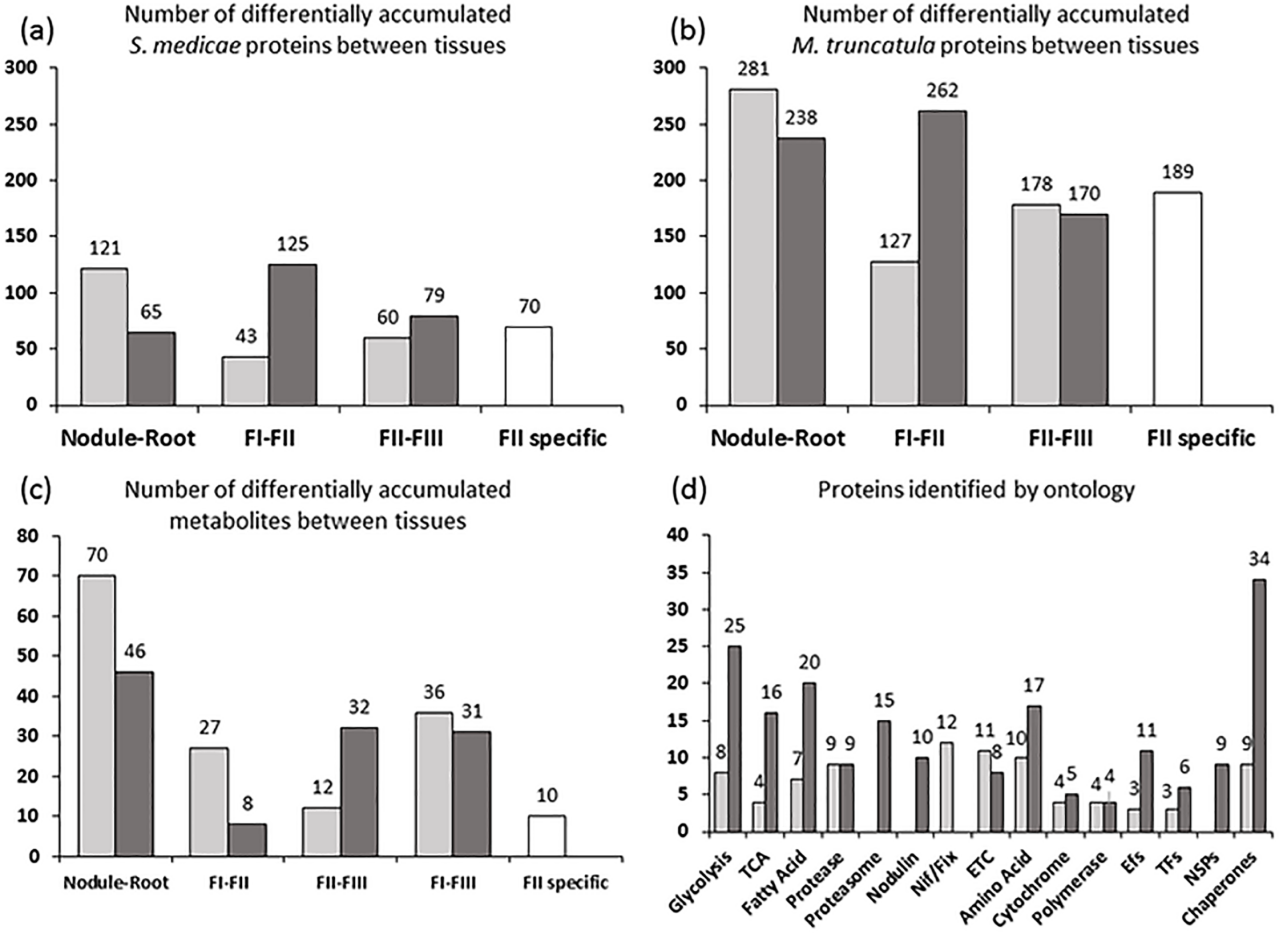
Summary of protein and metabolite profile data. (a)-(c) Number of proteins or metabolites showing significant intensity differences in pair wise tissue comparisons (*p-*value < 0.05). Light grey bars indicate the number of features with greater abundance in distal tissues, while dark grey bars indicate the number of proteins with greater abundance in proximal tissues. (d) Total proteins identified by ontology. Light grey and dark grey bars correspond to *S*. *medicae* and *M*. *truncatula* proteins, respectively. FI, Fraction I; FII, Fraction II; FIII, Fraction III; TCA, Tricarboxylic Acid Cycle; ETC, Electron Transport Chain; EFs, Elongation Factors; TFs, Transcription Factors; NSPs, Nodule specific proteins.

After confirming that our FI, FII, and FIII fractions accurately and reproducibly represent ZII, IZ, and ZIII, we compared changes in protein profiles of each tissue. Fig 3A and 3B shows the number of proteins with significant intensity differences between consecutive fractions (t-test, *p*-value < 0.05). As expected, *S*. *medicae* proteins were highly up-regulated in the whole nodule as compared to the *S*. *medicae* inoculated root. At the level of sub-nodule fractions, 75% of *S*. *medicae* proteins with significant differences between FI and FII were upregulated in FII, suggesting a large metabolic transition occurring in FII (IZ according to previous annotation). Similarly, more *M*. *truncatula* proteins changed significantly in FII than between any other sub-nodule fractions (Fig 3B). Our analysis identified 70 *S*. *medicae* and 189 *M*. *truncatula* proteins with significant differences between FII and the surrounding fractions FI and FIII (Fig 3A and 3B). These data provide further evidence of a coordinated and drastic metabolic change occurring in FII. Finally, the number of proteins identified in different ontological categories is illustrated in Fig 3C and displays the depth of our approach.

### Proteome analysis reveals coordinated changes during nodule development

#### Glycolysis is reciprocally regulated between symbionts as development proceeds

Proteome data from each tissue was used to examine coordination of carbohydrate metabolism between symbionts during development. The *S*. *medicae* phosphofructokinase (PFK) Smed_2106, an enzyme that regulates glycolysis, was 3-fold less abundant in FIII compared to FI, with an intermediate abundance in FII (*p-*value 0.04). Conversely, the *M*. *truncatula* 6-PFK MTR_070s0026 was 47-fold more abundant in FIII compared to FI. Other *M*. *truncatula* PFK isozymes showed a similar trend, with MTR_6g090130 increasing 8-fold from FI to FII and remaining abundant in FIII. Interestingly, another rate limiting glycolytic enzyme, pyruvate kinase (PK) (MTR_1g061630), was significantly 10 fold less abundant in FIII compared to FI (*p-*value 0.02). These two changes are likely to divert the flow of phosphoenolpyruvate away from away from NAD regeneration via pyruvate dehydrogenase and the plant TCA cycle, diverting carbon flow through phosphoenolpyruvate carboxylase (PEPC) (MTR_2g092930 & MTR_2g076670) which were 17-fold and 7-fold more abundant in FIII compared to FI (*p-*value 0.005 and 0.001, respectively). The pattern of increased *M*. *truncatula* PFKs and decreased *S*. *medicae* PFK from FI to FIII suggests a coordinated change in carbohydrate metabolism between symbionts. *M*. *truncatula* carbohydrate catabolism likely increased in FIII to produce C-4 dicarboxylic acids via PEPC in support of bacteroid metabolism. Decreased *S*. *medicae* PFK in FIII suggests a decreased use of carbohydrate carbon sources by bacteroids and further supports the role of C-4 dicarboxylic acids as their major carbon source [40,41].

#### Spatial localization of *S*. *medicae*'s transition to TCA dependent carbon metabolism

**Fig 4:**
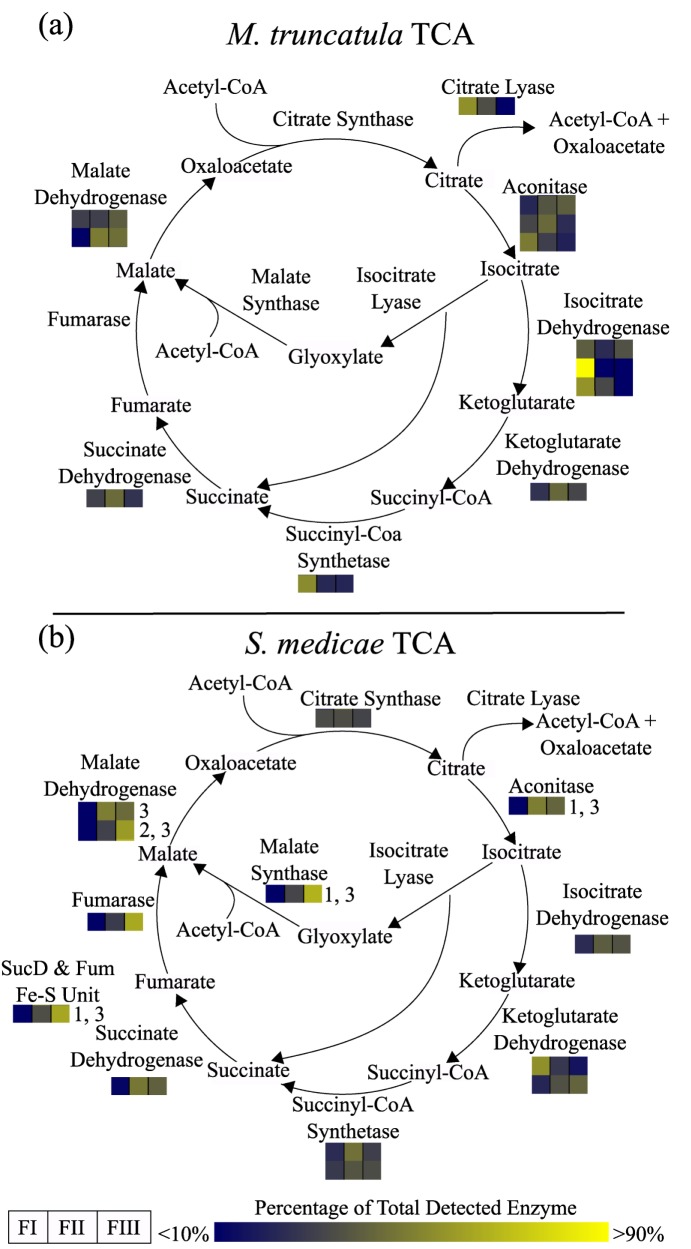
**Relative abundance of TCA cycle enzymes from (a) *M*. *truncatula* and (b) *S*. *medicae*.** Each heat map box corresponds to FI-FIII. The color for each box corresponds to the abundance of a protein expressed as a percentage of the total amount of that protein found in all three fractions.

Fig 4 displays the relative abundance of *S*. *medicae* and *M*. *truncatula* TCA cycle enzymes in each nodule fraction. Many *S*. *medicae* TCA enzymes, including aconitate hydratase (Smed_3083), succinyl-CoA Ligase (Smed_2942), and malate synthase (Smed_3270), and malate dehydrogenase (Smed_2944), were ≥2 fold more abundant in FII and FIII as compared to FI (*p-*value <0.05). These observations suggest *S*. *medicae* transitions to greater reliance on the TCA cycle in the interzone FII. With the noted exception of malate dehydrogenase, all other *M*. *truncatula* TCA cycle enzymes were unchanged or decreased from FI to FIII. These findings indicate *S*. *medicae* transitions from carbohydrate to dicarboxylic acid dependent metabolism in FII.

#### Coordinated changes in branched chain amino acid metabolism

MS identified 26 proteins involved in amino acid metabolism, 21 of which show significant differences in abundance between fractions (ANOVA, *p-*value <0.05). Of particular interest are proteins necessary for synthesis of branched chain amino acids (BCAAs), which are critical to SNF in closely related systems [42–44]. *S*. *medicae*'s 3-isopropylmalate dehydratase LeuC (Smed_3059) and ketol-acid reductoisomerase IlvC (Smed_1962) were 17- and 6-fold less abundant in FIII compared to FI, respectively (*p-*value <0.0001). These findings indicate that *S*. *medicae* BCAA synthesis decreases during bacteroid development, similar to patterns observed in other SNF systems.

Interestingly, a similar trend is observed in abundance patterns of the *M*. *truncatula* BCAA synthesis enzymes 2-isopropylmalate synthase (MTR_1g116500), 3-isopropylmalate dehydratase (MTR_1g045750), and ketol-acid reductoisomerase (MTR_4g079780), that show a 2 fold decrease in FIII compared to FI [45]. Despite these observations, however, our GC-MS metabolite profiling analysis showed that levels of leucine, isoleucine, and valine do not significantly change between fractions, suggesting that the 2-fold decrease in plant BCAA biosynthetic enzymes did not lead to decreased BCAA pools, or that alternative mechanisms exist to maintain BCAA pools between zones (S3 Table).

### Dynamic zone-specific accumulation of fatty acid biosynthesis and modification enzymes

Proteomic analysis identified 20 proteins involved in fatty acid (FA) metabolism whose abundance significantly changed between the three fractions (ANOVA, *p-*value < 0.05). The early steps of *S*. *medicae’s* FA biosynthesis are catalyzed by the enzymes FabB (Smed_3449) and Acetyl-CoA carboxylase subunits (Smed_0952 and Smed_0953), which were 15-, 3-, and 500-fold more abundant in FIII compared to FI (*p-*value<0.05). These observations suggest *S*. *medicae* requires increased FA production as SNF develops. The *M*. *truncatula* FA elongation enzyme 3-oxoacyl-ACP synthase (MTR_122s0008) was also more abundant in FIII compared to FI (*p-*value = 0.0001).

In addition to FA biosynthesis, we also observed fraction-specific differences in FA modifying enzymes of the lipoxygenase protein family. Lipoxygenases are known to alter gene expression, exhibit anti-microbial activity, and produce the plant hormone jasmonate [46]. *M*. *truncatula* lipoxygenases, annotated Lip1-5, differed in their patterns of accumulation across fractions. Lip1 and Lip2 were highly abundant in FI and decreased significantly across subsequent fractions. Conversely, Lip4 and Lip5 were most abundant in FIII and decreased 9- and 35-fold in FI, respectively. Lip3, however, was maximally abundant in FII, and decreased in abundance in the adjacent FI and FIII fractions (*p-*value = 0.01). These localizations suggest a distinct function for different lipoxygenases and modified FAs during nodule development. These changes in localization of FA biosynthesis and modifying proteins were consistent with the distribution of the corresponding mRNAs [23].

### Highly distinct metabolite profiles of SNF tissues

To provide additional insight into the physiology of nodule development, we generated primary metabolite profiles for each sub-nodule fraction using gas chromatography coupled to mass spectrometry (GC-MS). Our method successfully identified 160 metabolites, many of which showed a significant difference in abundance between different fractions (Fig 3C). These 68 compounds include 8 fatty acids, 18 sugars, 6 dicarboxylic acids, 11 amino acids, and 3 sterols. The transition zone FII contained 82 metabolites with an average intensity that was intermediate between FI and FIII. Fig 2C displays the PLS-DA scoreplot generated from these profiles and identifies metabolites whose abundance correlates with the various fractions [47]. Component one of the PLS-DA scoreplot accounts for 50.0% of the data variation, and was due primarily to differences between FI and FIII.

### Glycolysis and TCA metabolite abundances support proteome findings

The glycolytic intermediates fructose-6-phosphate and glucose-6-phosphate were 2- and 6-fold less abundant, respectively, in FIII compared to FI, indicating a greater need for glycolysis in the nitrogen fixing regions of the nodule (*p-*value <0.02). This was consistent with the observed increase of *M*. *truncatula* PFKs in FIII. Additionally, pyruvate was 2-fold less abundant in FIII than FI (*p-*value = 0.001). Furthermore, the intensity of malate significantly decreased in FIII and FII as compared to FI. The combination of decreased malate with increased levels of *S*. *medicae* dicarboxylic acid consuming enzymes, beginning specifically in FII, was consistent with a transition from glycolysis to TCA dependent metabolism. Altogether, the levels of sugar phosphates and dicarboxylic acids were consistent with the proteome changes observed, suggesting a net use of plant glycolysis up to PEP in FIII as compared to FI, accompanied by malate consumption via the TCA cycle in bacteroids.

## Discussion

### Sub-nodule protein profiles reveal coordinated changes in plant and bacterial metabolism during nodule development

Indeterminate nodules contain conspicuous developmental zones along their longitudinal axis, each of which represents a particular stage in the metabolic development of SNF. While many previous experiments have outlined aspects of SNF metabolism using protein and metabolite profiling methods, they have not focused on the coordinated changes that occur in each zone as the nodule develops. Transcriptomes have recently been generated for each zone using a closely related system, *M*. *truncatula-S*. *meliloti* [23]. Here, we describe a complementary study that applies a label free LC-MS technique to create protein profiles for whole-nodules, three sub-nodule fractions, and roots of the *M*. *truncatula-S*. *medicae* symbiosis. To yield additional insight about the consequences of the changes in protein abundance, we also collected GC-MS primary metabolite profiles from each sub-nodule fraction.

**Fig 5:**
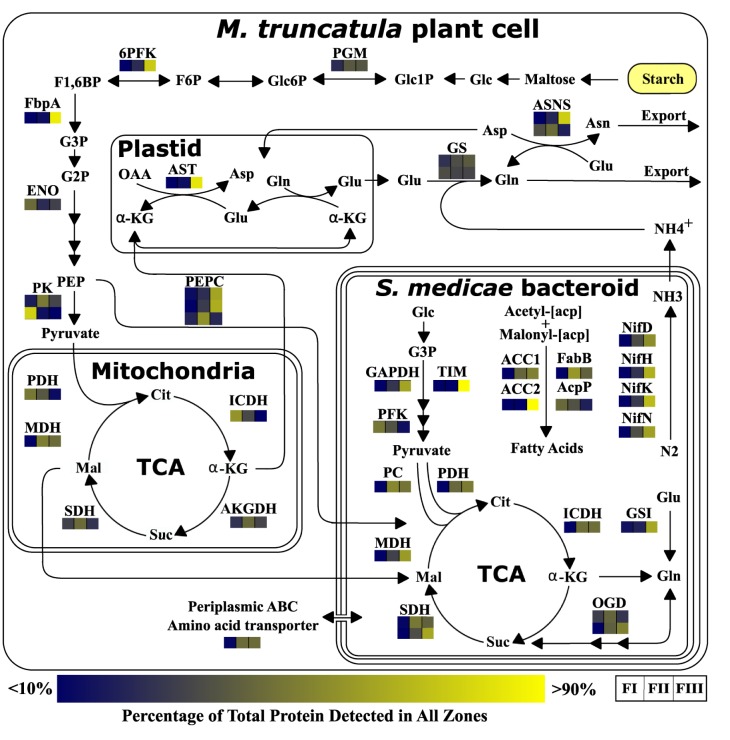
Comprehensive model of coordinated metabolism of carbohydrates, central carbon, fatty acids, amino acids, and others. 6PFK, 6 phosphofructokinase; ACC1, Acetyl-CoA carboxylase 1; ACC2, Acetyl-CoA carboxylase 2; AcpP, Acyl carrier protein; AKGDH, Alpha ketoglutarate dehydrogenase; Asn, Asparagine; ASNS, Asparagine synthase; Asp, Aspartate; AST, Aspartate transaminase; Cit, Citrate; ENO, Enolase; F1,6BP, Fructose 1,6-bisphosphate; F6P, Fructose 6-phosphate; FabB, 3-oxoacyl-(acyl carrier protein) synthase I; FbpA, Fructose-bisphosphate aldolase; FI, Fraction I; FII, Fraction II; FIII, Fraction III; G2P, Glyceraldehyde 2-phosphate; G3P, Glyceraldehyde 3-phosphate; GAPDH, Glyceraldehyde phosphate dehydrogenase; Glc, Glucose; Glc1P, Glucose 1-phosphate; Glc6P, Glucose 6-phosphate; Gln, Glutamine; Glu, Glutamate; GS, Glutamine synthetase; GSI, Glutamine synthetase I; ICDH, Isocitrate dehydrogenase; Mal, Malate; MDH, Malate dehydrogenase; NifD, Nitrogenase molybdenum-iron protein alpha chain; NifH, Nitrogenase reductase; NifK, Nitrogenase molybdenum-iron protein beta chain; NifN, Nitrogenase molybdenum-cofactor biosynthetic protein; OAA, Oxaloacetate; OGD, Oxoglutarate dehydrognease; PC, Pyruvate carboxylase; PDH, Pyruvate dehydrogenase; PEP, Phosphoenolpyruvate; PEPC, Phosphoenolpyruvate carboxylase; PFK, Phosphofructokinase; PGM, Phosphoglucomutase; PK, Pyruvate kinase; SDH, Succinate dehydrogenase; Suc, Sucrose; TCA, Tricarboxylic acid cycle; TIM, Triose phosphate isomerase; α-KG, alpha ketoglutarate.

The accuracy of our zone isolation method was validated using previously described biomarkers for each zone. Furthermore, the ability of our methods to capture distinct protein and metabolite profiles of the different fractions was shown using multivariate analysis including PCA and PLS-DA. We then demonstrated that we were able to follow important changes in relative abundance of plant and bacterial enzymes across the three sub-nodule zones with an emphasis on carbohydrate, central carbon, fatty acid, and amino acid metabolism. For each of these metabolic processes, the spatial patterns of sub-nodule protein abundance were largely in agreement with zone-specific transcript levels observed by Roux et al. (2014). Furthermore, the integration of metabolite profiles from each fraction corroborated many of our proteome observations. Fig 5 shows a metabolic pathway model that captures the dynamic and coordinated nature of multiple metabolic processes including glycolysis, TCA, fatty acid biosynthesis, and others. The data set in S2 Table would allow other pathways to be examined.

As SNF develops, multiple changes in fraction-specific protein profiles confirmed current paradigms of symbiosis. For example, the role of C-4 dicarboxylic acids as the primary carbon source for terminally differentiated bacteroids was supported by observed decreases in *S*. *medicae* glycolysis enzymes and increases in TCA enzymes from FI to FIII. These changes appear to be coordinated with increased accumulation of *M*. *truncatula* glycolysis enzymes from FI to FIII, likely to accommodate growing bacteroid use of dicarboxylic acids as their primary carbon source. The pattern of proteins involved in the transition of both plant and bacterial pathways to support the dicarboxylic acid dependency of bacteroids was localized to our equivalent of the IZ, FII, and therefore identified this metabolic shift as a novel characteristic of the IZ. It is interesting to note that *M*. *truncatula* pyruvate kinase (MTR_1g061630) was significantly less abundant in FIII compared to FI. This change was different than that of the *M*. *truncatula* PEPC isozymes, including MTR_2g092930 and MTR_2g076670, which both showed a significant 17- and 7-fold increase in FIII as compared to FI. This is consistent with the idea that regenerating the NAD+ needed to complete a glycolytic fermentation in the *M*. *truncatula* compartments was carried out by diverting phosphoenolpyruvate to oxaloacetate via PEPC and then to malate using malate dehydrogenase. Malate would then be fed to the bacteroids as a dicarboxylic acid source.

We were interested to see fraction-specific (i.e. zone-specific) abundance patterns of fatty acid biosynthetic (FAB) and modifying enzymes. The marked accumulation of *S*. *medicae* FAB proteins in FIII as compared to FI strongly suggests that the bacteria are producing fatty acids. Increased FA synthesis was likely caused by requirements for membrane growth characteristic of bacteroids or may be used as a sink for surplus reductant. This idea was supported by significant decreases in free FAs in FIII as compared to FI, including linoleic, lauric, stearic, and lignoceric acid, which we propose were being esterified to glycerol for incorporation into membranes. The fraction-specific patterns of multiple *M*. *truncatula* lipoxygenases further support the involvement of modified lipids in nodule development.

Understanding the changes in symbiont metabolism that occur during indeterminate nodule development has been the goal of many projects in the rhizobia research community. Early attempts at achieving this were limited to monitoring the location of one or a small set of gene products within the nodule via histological techniques. More recently, rhizobia mutants that cause nodules to become arrested at particular stages of development have been examined at the protein and transcript level. Advances in protein, transcript, and metabolite extraction methods, coupled with ever increasing instrument sensitivity is only now allowing us to observe the metabolic changes occurring within a wildtype indeterminate nodule. This work represents a substantial contribution toward understanding wild-type symbiont development at the protein and metabolite levels.

## Supporting information

S1 Table Proteome and metabolome dataset normalizations. (XLSX)

S2 Table Proteome dataset of root, FI-FIII, and nodule tissues. (XLSX)

S3 Table Metabolome dataset of root, FI-FIII, and nodule tissues. (XLSX)
